# Assessment of Lead and Cadmium Levels in Frequently Used Cosmetic Products in Iran

**DOI:** 10.1155/2013/962727

**Published:** 2013-09-23

**Authors:** H. Nourmoradi, M. Foroghi, M. Farhadkhani, M. Vahid Dastjerdi

**Affiliations:** ^1^Department of Environmental Health Engineering, School of Health, Ilam University of Medical Sciences, Ilam, Iran; ^2^Department of Environmental Health Engineering, School of Health, Hamadan University of Medical Sciences, Hamadan, Iran; ^3^Department of Environmental Health Engineering, Environment Research Center, School of Health, Isfahan University of Medical Sciences, Hezar Jerib Avenue, Isfahan, Iran

## Abstract

This study aims to investigate the content of lead and cadmium in most frequently used brands of cosmetic products (lipstick and eye shadow) in Iran. Fifty samples of lipstick (5 colors in 7 brands) and eye shadow (3 colors in 5 brands) were selected taken from large cosmetic stores in Isfahan (Iran) and lead and cadmium of them were analyzed. The results showed that the concentration of lead and cadmium in the lipsticks was within the range of 0.08–5.2 **µ**g/g and 4.08–60.20 **µ**g/g, respectively. The eye shadow samples had a lead level of 0.85–6.90 **µ**g/g and a cadmium level of 1.54–55.59 **µ**g/g. The content range of the heavy metals in the eye shadows was higher than that of the lipsticks. There was significant difference between the average of the lead content in the different brands of the lipsticks and eye shadows. Thus, the continuous use of these cosmetics can increase the absorption of heavy metals, especially Cd and Pb, in the body when swallowing lipsticks or through dermal cosmetic absorption. The effects of heavy metals such as lead can be harmful, especially for pregnant women and children. Therefore, effort must be made to inform the users and the general public about the harmful consequences of cosmetics.

## 1. Introduction 

Many thousands of years ago, the people of primary societies used various materials in order to beautify their faces [1]. Eye shadow and lipstick are a group of cosmetic products that have been most commonly used in various parts of the world. Many studies have reported that cosmetic products contain relatively high concentrations of heavy metals [2–7]. Kohl, a type of customary cosmetic product used for eyeliner in the Middle East, contains more than 50% of lead [5]. On the basis of a study, it was determined that the concentration of blood lead in a seven-month baby was 39 **µ**g/dL because of the consumption of kohl [8]. The use of leaded eye cosmetics has been observed to be strongly correlated with elevated blood lead levels [9]. Lipstick and eye shadow have various components including antioxidants, pigments, waxes, oils, and inorganics such as silica, TiO_2_, copper powder, and aluminum powder, bronze powder. Heavy metals are found in ingredients that naturally contain heavy metals or are polluted with them during production or by containers [7, 10]. Some toxic elements including heavy metals (lead, cadmium, etc.) have been found as impurities in pigments of lipsticks, eye shadows, and face powders [4, 6]. Al-Saleh et al. showed that heavy metals (e.g., lead) can be absorbed by children's and women's skin through using cosmetic products [2, 3]. 

Contact with low concentration of lead can cause disorders such as behavioral abnormalities and decreased learning and hearing and can result in adverse effects on central nervous system, reproductive system, hematopoiesis, anemia, and hepatic and renal systems [1, 11–13]. More than 90% of lead absorbed by human is concentrated in the bones with a half-life of greater than 20 years [14]. Cadmium is one of the major heavy metals found in some natural colors and inorganic pigments of cosmetic products [10]. Cadmium can concentrate primarily in kidney, bones, and teeth, and long-term contact with it causes growth retardation of rat fetus and teratogenic consequences and weight loss [1, 15]. The half-life of cadmium in human is determined to be 10–35 years [15]. International Agency for Research on Cancer (IARC) has categorized lead and cadmium in group 2A of carcinogen [15]. It is well proven that lead and cadmium can pass the placenta for the period of pregnancy and have been related to uterus fetal death before the proper time of delivery and low birth weight [16, 17]. US Food and Drug Administration (FDA) has shown that the average concentration of lead in 400 samples of lipsticks was 1.11 **µ**g/g [7]. In 2007, a study on the lead concentration of 33 brands of lipsticks showed that 61% of tested lipsticks have measurable lead (0.03–0.65 **µ**g/g) [18]. The US FDA has not set a value as an acceptable level for lead and cadmium in cosmetic products including lipstick. But many studies have used the FDA lead limit for candy (0.1 **µ**g/g) as a permissible standard for lipsticks [19]. It has been determined that women unintentionally swallow 4 pounds of lipstick during their life [20]. Iran among the Middle East countries is the third biggest user of cosmetic products [21]. With regard to this issue and adverse effects of consuming cosmetic products in Iran, the aim of this study was to investigate the concentration of lead and cadmium in the most commonly used brands of cosmetic products (lipstick and eye shadow).

## 2. Materials and Methods

### 2.1. Samples

Cosmetic products including the most commonly used brands of lipstick (35 samples: 5 colors in 7 brands) and eye shadow (15 samples: 3 colors in 5 brands) were purchased from large cosmetic stores in Isfahan, Iran. The colors studied for each brand of lipstick were orange, black grown, pink, violet, and copper and for each brand of eye shadow were blue, green, and copper.

### 2.2. Samples Preparation and Analysis

One gram of each sample was placed into a 100 mL Pyrex glass beaker and digested with 5 mL of concentrated nitric acid (Merck, 99.99%) on a hot plate at 80°C until it dried. The digestion process was repeated twice. Then, 1 mL of concentrated H_2_O_2_ was added in order to oxidize completely the organic matter of residues. The residual material was diluted with deionized water to the final volume of 50 mL. The solution was then filtered by Whatman filter (Merck, 0.45 **µ**m) [9]. The concentration of the metal ions in the solution was determined by a Graphite furnace atomic absorption spectrometry (GFAAS, Model AAnalyst 300). The detection limit of the GFAAS was 0.1 ppb for both metals. The metal ion content in the cosmetic products was reported as microgram per gram (**µ**g/g) on the basis of wet weight. Statistical analysis (SPSS-16, one-way ANOVA) was conducted to determine the relation of metal ion concentration among the cosmetic products. The *P* value of 0.05 was considered as significant.

## 3. Results and Discussion

Fifty samples of lipstick (35 samples) and eye shadow (15 samples) were investigated in this study. The samples analyzed showed that lead and cadmium were detected in all brands of the cosmetics with varying concentrations (Table 1). As seen, the concentration of lead and cadmium in the lipsticks was within the range of 0.08–5.2 **µ**g/g and 4.08–60.20 **µ**g/g, respectively. The eye shadow samples had also a lead level of 0.85–6.90 **µ**g/g and a cadmium level of 1.54–55.59 **µ**g/g.

Based on the results illustrated in Table 1, cadmium content in both cosmetic products was higher than lead content (*P* < 0.04). There was a significant difference between the average of the lead content in the different brands of the lipsticks (*P* = 0.018). Similar results were obtained for the different brands of eye shadows (*P* = 0.02). The statistical analysis also confirmed the meaningful difference between cadmium content for various brands of lipstick (*P* < 0.03), but this result did not attain for the eye shadows (*P* > 0.05). Because of the lack of governmental and international rules associated with the maximum permissible content of lead in cosmetics, the Campaign for Safe Cosmetics (CSC) has set 0.1 **µ**g/g for lead in cosmetics such as lipstick. This rule has been assigned on the basis of the maximum allowable lead concentration in candy, because it has been assumed that lipstick may be directly taken in via the mouth [18]. This standard value is much lower than that of the lead level in most lipsticks in various studies [9, 22–24]. Therefore, it is seen that this is not a valid standard because candy is used for ingestion, but lipstick is externally consumed on the lips and it may be inadvertently ingested [25]. 

US Food and Drug Administration (USFDA) has suggested that the concentration of some heavy metals such as nickel, cobalt, and chromium in color additive cosmetics should be less than 170 **µ**g/g and that of lead should be less than 20 **µ**g/g [25, 26]. As shown in Table 2, the content of lead in the studied cosmetic products was much lower than 20 **µ**g/g, which meets the US FDA regulation.

In comparison with other sources such as water, air, and food, daily exposure to heavy metals from cosmetics has been considered as a negligible source for human. Nevertheless, because of the cumulative properties of heavy metals in the body during life time, cosmetics can be regarded as a substantial source of the metals [9, 27]. Even under the best producing methods in the factories, the existence of heavy metals in cosmetics is inevitable [25]. Therefore, in order to diminish the adverse health effects of heavy metals, cosmetics producers must use such ingredients as color additives in their cosmetics to meet FDA's requirements.

The use of leaded cosmetics such as lipstick and eye shadow has been found to severely affect human beings, especially pregnant women, young children, and fetus. In pregnant women lead can easily cross the placenta and produce congenital lead poisoning [9, 28]. Many studies have proven the relation between consuming leaded cosmetics (lipstick and eye shadow) and elevated blood lead levels [23, 29]. It has been observed that the blood lead level of eye cosmetics consumers in Pakistan, India, and Saudi Arabia in comparison with nonconsumers was threefold [9]. Blood lead levels under 10 **µ**g/dL may damage neurobehavioral development in children. It has been proved that by increasing one microgram lead per deciliter (**µ**g Pb/dL) of blood, the intelligence quotient (IQ) of children is reduced by 0.25 points [30]. Lead has also been related to infertility and miscarriage [23]. The continuous use of cosmetics could have adverse effects on the ocular system [9]. These harmful effects can be caused by skin contact [31].

The studies conducted by Nnorom et al., Khalid et al., and Tsankov et al. showed that the values of lead in all tested brands of lipsticks were up to 41.1 **µ**g/g, 87–123 **µ**g/g, and 0.286–6.234 **µ**g/g, respectively [9, 22, 24]. The results of the present study showed that the lead content in the lipstick samples was generally lower than that of the mentioned studies. In this study, the content of lead in the eye shadows was also lower than 10 **µ**g/g. Al-Saleh et al. showed that the lead levels in 15 different colors of 8 brands of eye shadows were in the range of 0.42–58.7 **µ**g/g [23]. Sainio et al. also reported that the lead content in 25 brands of eye shadows was up to 16.8 *μ*g/g [6]. The concentration of lead in the eye shadows in the present study was also lower than the above-mentioned studies. 

Tables 2 and 3 also illustrate that the content range of the heavy metals in the eye shadows was higher than that of the lipsticks (*P* < 0.001 for lead and *P* = 0.04 for cadmium).  Table 3 also presents the concentration range of the cadmium in the cosmetic products. As shown, 29.4% and 66.7% of the lipstick and eye shadow samples had cadmium content more than 20 **µ**g/g, respectively.

The presence of cadmium in the samples can also have harmful effects on the human body. Small amounts of cadmium may result in heart disease, hypertension, kidney and liver damage, and weakened immune system [32]. 

Health Canada has recommended that the maximum cadmium concentration is 3 **µ**g/g in cosmetics. Therefore, a trace amount of cadmium is not safe [33]. The results of this study (Table 3) showed that the amount of cadmium in the cosmetics under study was much higher than the standard.

Nnorom et al. reported that the average of cadmium levels in several facial cosmetics (eye cosmetics, lipsticks, and lip gloss) was approximately 1 **µ**g/g [9]. Khalid et al. also showed that cadmium content in all brands and colors of lipsticks was within the range of 0.200–0.500 **µ**g/g [22]. The amount of cadmium in the present study in comparison with that of the above study was far more. 

Table 4 shows the heavy metals content in various colors of the cosmetic products. As seen, although the lipsticks with copper color have maximum levels of lead (2.21 **µ**g/g) and cadmium (27.20 **µ**g/g), the statistical analysis did not confirm these differences (*P* > 0.2).

The findings (Table 4) also confirmed that eye shadows with golden color have a higher concentration of cadmium (*P* = 0.043), but there was no significant difference between lead content of golden color eye shadow of the other various colors (*P* = 0.92), while Al-Saleh et al. reported that eye shadows with darker color pigments in their formulation have the highest values of heavy metals [23]. Khalid et al. also indicated that the highest concentration of heavy metals was in lipsticks with dark brown and shocking pink colors, and cosmetics with pink color had the lowest metal contents [22].

## 4. Conclusion

The concentration of lead in the cosmetics under study was lower than that of FDA standards, and the cadmium content in the samples was relatively high. The continuous use of these cosmetics can increase the absorption of heavy metals especially Cd and Pb into the body when swallowing lipsticks or through dermal cosmetic absorption. The effects of heavy metals such as lead in cosmetics can be harmful. Therefore, effort must be made to inform the users and the general public especially pregnant women and children of the harmful consequences of cosmetics.

## Figures and Tables

**Table 1:** Concentration of lead and cadmium in different brands of cosmetic products.

Brand	Lead content (**µ**g/g of wet weight)				Cadmium content (**µ**g/g of wet weight)			
	Min	Max	St. dev.	Average	Min	Max	St. dev.	Average
Lipstick								
1	0.48	3.00	0.96	2.03	5.84	17.97	5.06	14.82
2	0.08	2.40	0.92	0.93	7.12	40.52	13.69	16.41
3	0.36	0.93	0.26	0.58	12.49	32.68	8.99	19.75
4	0.05	4.20	1.67	1.47	4.08	59.96	26.42	26.66
5	0.26	3.12	1.12	1.18	5.60	38.08	12.62	17.29
6	0.08	1.49	0.65	0.97	11.33	14.68	1.33	12.99
7	1.80	5.20	1.59	3.36	28.46	60.20	12.92	37.96
Eye shadow								
1	4.47	6.90	1.22	1.55	20.72	50.62	15.30	33.72
2	2.27	2.70	0.22	2.50	17.62	55.59	21.75	30.47
3	3.74	6.00	1.15	5.00	1.54	39.18	19.13	22.35
4	0.85	1.63	0.42	1.15	6.42	41.32	19.44	28.82
5	2.57	4.57	1.07	3.35	16.75	24.01	3.90	21.23

**Table 2:** Concentration range of lead (**µ**g/g) in the cosmetic products.

Concentration range (**µ**g/g)	Frequency percent (%)	
	Lipstick	Eye shadow
<1	47.0	13.3
1-2	20.6	6.7
2–5	17.7	53.3
5–10	14.7	26.7

**Table 3:** Concentration range of cadmium (**µ**g/g) in the cosmetic products.

Concentration range (**µ**g/g)	Frequency percent (%)	
	Lipstick	Eye shadow
<10	14.7	13.3
10–20	55.9	20.0
20–30	2.9	33.3
30–40	14.8	13.3
40–50	5.9	6.8
>50	5.8	13.3

**Table 4:** Concentration of lead and cadmium in different colors of cosmetic products.

Color	Lead content (**µ**g/g of wet weight)				Cadmium content (**µ**g/g of wet weight)			
	Min	Max	St. dev.	Average	Min	Max	St. dev.	Average
Lipstick								
Black brown	0.10	5.20	1.87	1.55	1.52	60.20	19.16	18.21
Copper	0.48	4.97	1.74	2.21	5.84	43.36	16.26	27.20
Orange	0.10	2.68	1.11	1.07	10.76	59.90	17.57	25.54
Pink	0.32	3.20	0.75	1.37	4.08	31.90	10.43	13.30
Violet	0.23	3.12	1.04	1.49	12.42	28.46	5.45	17.26
Eye shadow								
Blue	0.97	6.00	1.98	3.25	16.75	55.59	14.67	33.44
Green	1.63	6.90	2.01	3.57	1.54	22.92	9.42	13.96
Golden	0.85	5.60	2.02	3.76	17.62	50.62	13.45	34.55
